# The Use of Two-Dimensional Shear Wave Elastography in People with Obesity for the Assessment of Liver Fibrosis in Non-Alcoholic Fatty Liver Disease

**DOI:** 10.3390/jcm10010095

**Published:** 2020-12-29

**Authors:** Ritesh Chimoriya, Milan K Piya, David Simmons, Golo Ahlenstiel, Vincent Ho

**Affiliations:** 1 School of Medicine, Western Sydney University, Campbelltown, NSW 2560, Australia; r.chimoriya@westernsydney.edu.au (R.C.); m.piya@westernsydney.edu.au (M.K.P.); da.simmons@westernsydney.edu.au (D.S.); g.ahlenstiel@westernsydney.edu.au (G.A.); 2 Camden and Campbelltown Hospitals, Campbelltown, NSW 2560, Australia; 3 Storr Liver Centre, Westmead Institute for Medical Research, University of Sydney, Westmead, NSW 2145, Australia; 4 Blacktown Hospital, Blacktown, NSW 2148, Australia

**Keywords:** 2D-shear wave elastography, transient elastography, obesity, non-alcoholic fatty liver disease, liver fibrosis

## Abstract

Obesity is associated with significant comorbidities, including non-alcoholic fatty liver disease (NAFLD). Given its potential to progress to advanced liver disease, monitoring the extent and progress of liver fibrosis and assessing its fibrosis stage are essential. Although liver biopsy is considered to be the gold standard for liver fibrosis staging, it is an invasive procedure with risk of complications. Considering the rising prevalence of obesity and NAFLD globally, developing non-invasive diagnostic methods is a priority. Transient elastography (TE) is increasingly being used to assess the severity of liver disease. However, in the presence of severe obesity, the increased thickness of subcutaneous adipose tissue and changes in anatomy may affect its diagnostic accuracy. Two-dimensional shear wave elastography (2D-SWE) assesses the liver stiffness in real time along with simultaneous anatomic B-mode ultrasound imaging and allows selection of the region of interest. This would suggest that 2D-SWE has several advantages over TE in patients with severe obesity. The purpose of this review is to examine the current literature addressing the use of 2D-SWE in the assessment of liver fibrosis in patients with NAFLD. This review also examines the evidence on the use of 2D-SWE in patients with obesity and NAFLD and compares it to TE as a novel and non-invasive method of assessing liver fibrosis.

## 1. Introduction

Over one-third of the world’s population today are either overweight or obese [1,2]. If recent secular trends continue, it is estimated that about 38% and 20% of the world’s adult population will be overweight and obese, respectively, by 2030 [3]. The prevalence of severe obesity (body mass index (BMI) >40 kg/m^2^ or >35 kg/m^2^ with a comorbidity) is also rapidly increasing [4], with current rates greater than 9% in many countries [5,6]. Severe obesity may cause a substantial reduction in life expectancy, as it is estimated that median survival is reduced by eight to ten years for people with a BMI of 40–45 kg/m^2^ compared to a BMI of 22.5 kg/m^2^ [7].

Non-alcoholic fatty liver disease (NAFLD) has become the most common liver disease and the leading cause of chronic liver disease worldwide [8,9]. NAFLD is strongly linked to obesity [10,11,12], with a reported prevalence of approximately 80% in people with obesity and only 16% in people with a normal BMI and no known metabolic risk factors [10,13]. Moreover, Asian and Hispanic ethnic groups are at a greater risk of NAFLD as compared to Caucasian and Afro-Caribbean ethnic groups, which could be attributed to metabolic factors, genetic predisposition, and the environment [14]. Recently, metabolic- associated fatty liver disease (MAFLD) was proposed as a more appropriate nomenclature than NAFLD, as it precisely reflects the current understanding of fatty liver diseases that are associated with metabolic dysfunction [15].

NAFLD is characterised by liver steatosis where >5% of hepatocytes are infiltrated with fat in patients with no history of alcohol abuse and no competing aetiologies for hepatic steatosis [9]. NAFLD represents a spectrum of disorders ranging from simple hepatic steatosis or non-alcoholic fatty liver (the most common form) to non-alcoholic steatohepatitis (NASH), which is histologically characterised by hepatocyte injury, inflammation, and variable degrees of fibrosis that can further lead to advanced fibrosis and cirrhosis [16,17,18]. Approximately 10–30% of patients with simple steatosis develop NASH [9,19], 5–10% of those patients develop liver cirrhosis within five years [9,20], and just over 1 in 8 patients with liver cirrhosis develop hepatocellular carcinoma within three years [21]. Given its potential to progress to advanced liver disease, the accurate diagnosis of those with NASH is critical. Moreover, monitoring the extent and progress of liver fibrosis and assessing its fibrosis stage or the presence of cirrhosis are essential in the management of patients with liver disease [22,23].

Development of non-invasive methods of measuring liver fibrosis as an alternative to the gold standard liver biopsy has been an important area of clinical research. Among non-invasive methods, transient elastography (TE) is increasingly being used for the assessment of fibrosis in liver diseases [24,25]. Recently, two-dimensional shear wave elastography (2D-SWE) has emerged as a novel non-invasive diagnostic tool that could be particularly useful for people with obesity [24,26]. The purpose of this review is to examine the current literature addressing the use of 2D-SWE in the assessment of liver fibrosis in patients with NAFLD. This review also examines the evidence on the use of 2D-SWE in patients with obesity and NAFLD and compares it to TE as a novel and non-invasive method of assessing liver fibrosis.

## 2. Assessment of Liver Fibrosis in NAFLD

Traditionally, liver biopsy is considered to be the gold standard for the staging of liver fibrosis as it provides precise diagnostic information on necro-inflammation and levels of steatosis [27,28]. However, liver biopsy is a potentially painful and invasive procedure that can result in complications such as bleeding, especially in those patients with coagulopathy and thrombocytopenia from advanced liver disease [23,27,29]. Further the limitations of liver biopsy include patient refusal, targeted sampling error, interobserver variation in staging, heterogeneity of liver fibrosis, and limited sampling range [27,28,30].

The meta-analysis of histological data in viral hepatitis (METAVIR) scoring system by Bedossa et al. [31] is the most commonly used tool to evaluate the severity of fibrosis. It integrates five fibrosis stages: F0 (no fibrosis), F1 (mild fibrosis, portal fibrosis without septa), F2 (significant fibrosis, portal fibrosis with few septa), F3 (severe fibrosis, numerous septa without cirrhosis), and F4 (cirrhosis). On the other hand, the Brunt system is a useful benchmark for diagnosing NASH, while the NASH Clinical Research Network (CRN) scoring system is one of the most validated systems currently available [32,33].

Currently, several non-invasive diagnostic methods are available as an alternative to liver biopsy. Simple non-invasive scoring systems such as the NAFLD fibrosis score [34] have been developed to distinguish the severity of fibrosis in patients with NAFLD. It incorporates age, BMI, aspartate aminostransferase (AST), alanine aminotransferase (ALT), platelet count, blood glucose levels, and albumin levels. Angulo et al. [34] suggested that by applying the NAFLD fibrosis score, a liver biopsy to determine the severity of fibrosis can be avoided in approximately 75% of patients with NAFLD. Conventional ultrasonography (US) is the most common technique used for the assessment of morphological and structural changes to the liver in clinical settings [35,36]. Although conventional US is useful in evaluating cirrhosis, it has a lower sensitivity for diagnosing early stages of liver fibrosis [37,38].

### Non-Invasive Diagnostic Methods—Elastography

Various non-invasive diagnostic methods have been developed based on the assessment of liver stiffness measurement (LSM), which is a promising surrogate biomarker of liver fibrosis stage [37]. Among the elastography techniques available, magnetic resonance elastography (MRE), a magnetic resonance imaging (MRI)-based technique, has shown promising results [8,39]. However, MRE has several disadvantages which have limited its use in clinical practice and in people with obesity. These include high cost, longer examination times, failure to perform in a liver with iron overload due to low hepatic signal, and the MRE machine not being able to accommodate individuals with obesity [25,33,39]. Various US-based elastography techniques have also been developed, including shear wave elastography, which can be further classified into transient elastography (TE), point shear wave elastography (pSWE), and two-dimensional shear wave elastography (2D-SWE) [8,40]. While the biopsy sample represents 1/50,000 of the total liver mass, US-based elastography measures the liver stiffness or elasticity by assessing a liver volume that is at least 100 times larger than the biopsy sample [39,41].

TE was the first commercially available US-based elastography method developed for LSM [24,25]. In TE, the speed of mechanically generated shear waves at the surface of the skin is estimated to the assess the liver stiffness [24,42]. TE has shown an excellent diagnostic performance for detecting advanced fibrosis and cirrhosis in patients with NAFLD [43,44]. However, given that obesity is a major risk factor for NAFLD or NASH, TE may not provide an accurate LSM in patients with obesity [44,45]. As the fatty thoracic belt attenuates both ultrasound and elastic waves, TE using the standard M probe can result in higher failure rates in people with obesity [23,46,47]. Addressing this limitation, the XL probe was developed specifically for patients with a BMI >30 kg/m^2^, which has reduced the failure rates in people with obesity [23,48]. However, evidence is lacking in people with severe obesity, where increased thickness of subcutaneous adipose tissue and changes in anatomy may further affect its diagnostic accuracy [45,48].

2D-SWE and pSWE are more recently developed non-invasive methods to assess liver stiffness with several advantages in their applicability to people with obesity [24,25,26]. 2D-SWE was first introduced in 2005 on a diagnostic imaging device known as Aixplorer (SuperSonic Imagine, Aix-en-Provence, France) [42,49]. 2D-SWE is incorporated onto a conventional ultrasound diagnostic imaging device, allowing both morphological ultrasound liver examination and quantitative elastography assessment of liver fibrosis simultaneously [49]. Various high-end ultrasound systems that utilise 2D-SWE for the assessment of liver stiffness are commercially available, including those manufactured by Philips Healthcare and Siemens Healthcare [25]. 2D-SWE estimates the liver stiffness by measuring the speed of acoustically generated shear wave propagation in the tissue [41,50]. Liver stiffness is assessed in real time as the shear waves are generated by ultrasound pulses along with simultaneous anatomic B-mode ultrasound imaging [41,51]. 

## 3. Principle of 2D-Shear Wave Elastography

**Figure 1:**
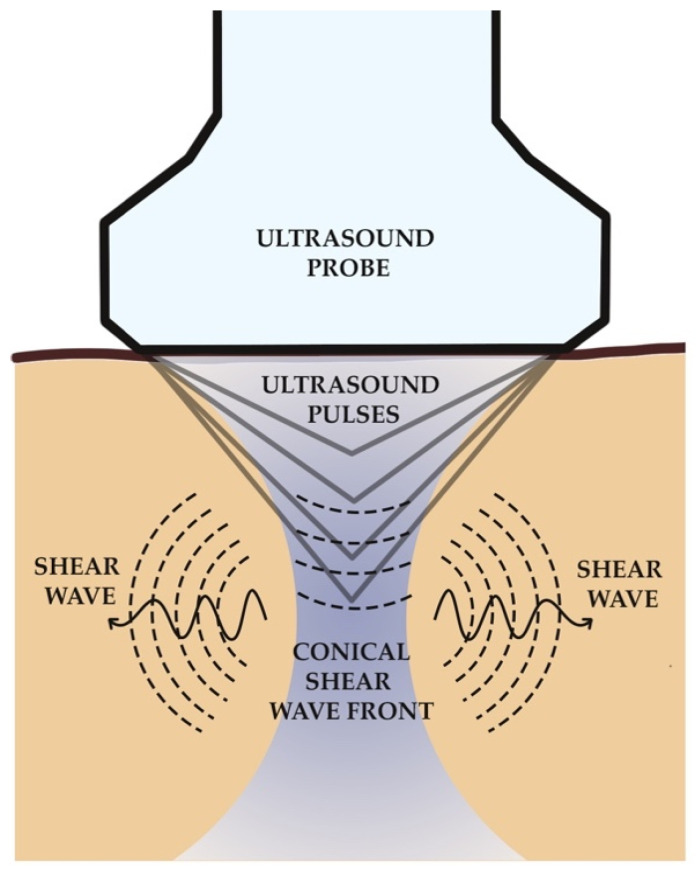
Basic principle of shear wave elastography (SWE). Ultrasound probe produces a localised radiation force deep in the tissue of interest, inducing a shear wave that propagates from this focal point. Numerous focal points are generated simultaneously in a line perpendicular to the skin surface. A conical shear wave front is created that sweeps the image plane on each side of the focal point. Adapted from Sigrist et al. [54] and Early et al. [55].

Shear waves are transverse waves that are generated when a directional force is applied to a tissue, causing shear deformation [52]. The principle behind the interpretation of 2D-SWE is that shear waves produced by a focused ultrasound beam are directly associated with the liver stiffness from where they are generated [26,39]. The shear waves are not generated on the surface of the body but near the region of interest (ROI) in the liver parenchyma [26]. The ultrasound probe of the device produces a localised radiation force deep in the tissue of interest, inducing a shear wave that propagates from this focal point. In a line perpendicular to the surface of the skin, numerous focal points are generated simultaneously; hence, creating a conical shear wave front that sweeps the image plane on each side of the focal point [49]. An illustration of this mechanism is shown in Figure 1. In 2D-SWE, the acoustic radiation force focus is swept down the acoustic axis faster than the shear wave speed, which generates tissue displacements (tens of μm) at all positions along the axis simultaneously. This creates cone-shaped shear waves travelling away from the push line that spreads less and decays less rapidly with distance, compared to that from a single pushing focus [53].

From a physics point of view, elastography assesses tissue elasticity, which is the tendency of the tissue to resist deformation when a force is applied or to resume to its original shape when the force is removed [54]. Elastography aims to quantitatively image the Young’s modulus, which is the physical parameter that corresponds to stiffness and has two major advantages: Young’s modulus is ideal for characterisation of different tissues with an exceptional contrast, as it exhibits significant variations between different biological tissues; and it characterises tissue stiffness, which has relevant diagnostic value and is precisely the quantitative reproduction of a clinician’s palpation [56]. Correspondingly, qualitative and quantitative estimates of the tissue elasticity are attained through the measurement of shear wave speed [54]. Subsequently, the ultrasound system monitors the shear wave propagation and measures its velocity, which is presented in meters per second (m/s) or in Young’s modulus kilopascals (kPa) [26,57].

In 2D-SWE, as tissue displacement occurs at multiple points using acoustic radiation force impulse, high frame rate imaging readily detects the resultant shear wave front. This is used to monitor shear wave propagation at multiple points in the image in real time [53,58]. UltraFast Imaging is used to capture the progression of shear wave at up to 20,000 images per second through the rapid acquisition of ultrasound images that take only a few milliseconds [49]. Shear waves are only generated at low frequencies (10 Hz to 2000 Hz) as they are absorbed by tissues at higher frequencies [56]. Moreover, shear waves propagate more slowly (1–10 m/s), which is why a high-speed acquisition is required to capture the shear waves [49,58]. Tissue displacements induced by the shear wave can be measured by comparing two consecutive ultrasound images and estimating the shear wave propagation speed.

**Figure 2:**
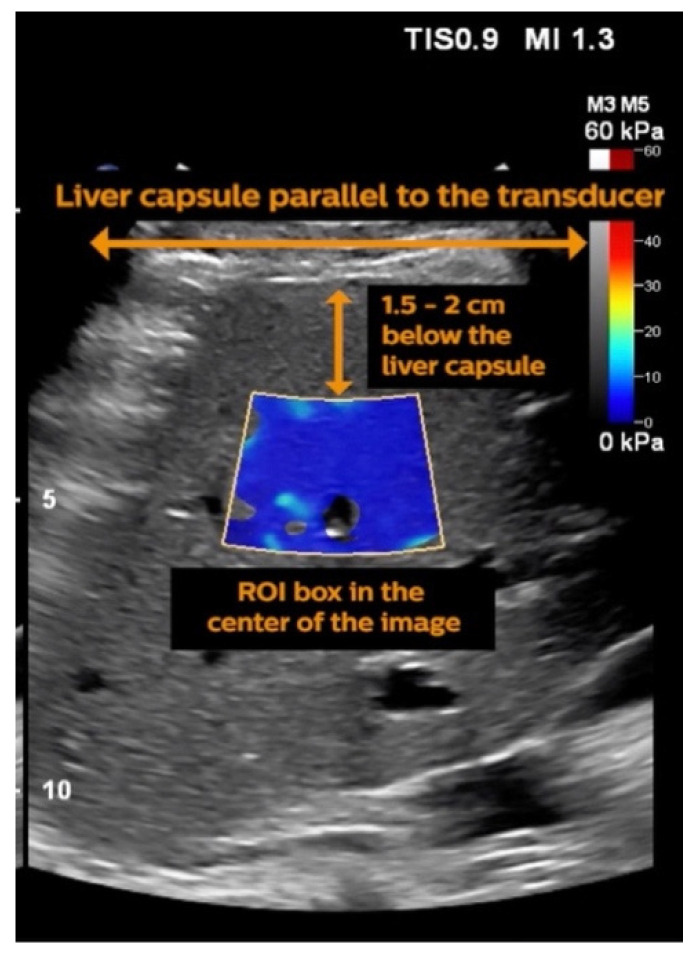
Example of results provided by 2D-SWE along with a 2D colour map and region of interest (ROI). The liver capsule is parallel to the transducer surface, and the ROI is positioned about 1.5–2 cm below the liver capsule. Adapted with permission from Philips Electronics Australia Limited [59].

Elastogram, a quantitative elasticity image, is displayed as a 2D colour map, where each colour codes either shear wave speed (m/s) or elasticity (kPa) as quantitative results [58]. Red and blue colours represent stiffer and softer tissues, respectively [8]. The real- time tissue stiffness colour maps are accompanied by an anatomic reference grey scale or B-mode image (Figure 2) [49,58]. Maximum elastogram size can range from 2 to 3 cm of side length using a linear probe to 9 × 4 cm using a convex probe [53,58]. This real-time imaging mode enables quantitative measurements by positioning one or more regions of interest (ROI—also known as Q-Box) [42,49]. The measurements should be performed on the right liver lobe about 1.5–2 cm below the liver capsule [25,59]. The ROI can be adjusted to variable sizes (3–700 mm^2^), and the measurements provided are the mean, standard deviation, and the minimum and maximum elastography values [49]. This may be particularly useful in people with severe obesity where the subcutaneous adipose tissue layer may be thick and the anatomy distorted.

## 4. Diagnostic Performance of 2D-Shear Wave Elastography

Several studies have reported a good diagnostic performance of 2D-SWE for the diagnosis of various stages of fibrosis in patients with NAFLD (Table 1). Review of the current literature shows that exclusive focus on people with obesity is limited. The studies have elucidated that obesity may influence evaluation performance of LSM [60] and is associated with a decline in area under receiver operating characteristics curve (AUROC) values [4]. Nonetheless, good diagnostic performance was observed even in the presence of obesity and severe obesity.

Takeuchi et al. [61] evaluated the accuracy of 2D-SWE in diagnosing fibrosis in patients with biopsy-proven NAFLD (mean BMI 29.2 kg/m^2^). The AUROC values in diagnosing ≥F1, ≥F2, ≥F3, and F4 were 0.82, 0.75, 0.82, and 0.90, respectively. The authors suggest that although a few studies have investigated the use of 2D-SWE in NAFLD patients, it is a useful tool for estimating the severity of liver fibrosis in this cohort. Similarly, a meta-analysis was conducted by Herrmann et al. [62] across 51 centres globally to assess the value of 2D-SWE for liver fibrosis staging in 156 patients with NAFLD (mean BMI 31.2 kg/m^2^) using liver biopsy as a reference. The diagnostic performance of 2D-SWE was good for the diagnosis of significant fibrosis (≥F2; AUROC = 0.86) and excellent for the diagnosis of severe fibrosis (≥F3; AUROC = 0.93) and cirrhosis (F4; AUROC = 0.92). The optimal cut-off points for diagnosing significant fibrosis and cirrhosis were found to be 7.1 and 13.0 kPa, respectively.

A recent study by Jamialahmadi et al. [4] assessed the diagnostic accuracy of 2D-SWE for detecting NAFLD as compared to the gold standard liver biopsy in participants with severe obesity (mean BMI 45.5 kg/m^2^). The success rate of 2D-SWE was 97.3% (108 of 111 patients), and failure in 3 patients was due to the presence of excessive subcutaneous adipose tissue. The AUROC values for 2D-SWE were 0.77, 0.72, 0.77, and 0.70 for ≥F1, ≥F2, ≥F3, and F4, respectively. The authors suggest that the decline in the AUROC values may be attributed to thick subcutaneous adipose tissue, which can interfere with the transmission of mechanical beam and ultrasound waves. The optimal cut-off values of 2D-SWE for ≥F2 and F4 were 6.6 and 6.8 kPa, respectively. In this study, higher BMI and waist circumference were found to decrease the accuracy of 2D-SWE. However, the authors conclude that 2D-SWE can be a feasible option for assessing liver fibrosis in individuals with severe obesity.

A recent meta-analysis conducted by Fu et al. [60] evaluated the diagnostic value of 2D-SWE in the assessment of hepatic fibrosis. The authors suggest that several factors, including the number of measurements, liver volumes, fibrosis stage, gamma-glutamyltransferase (GGT), serum albumin, and patient’s conditions, such as overweight or obesity and/or other complications, may influence the evaluation performance for LSM. In regards to the applicability of 2D-SWE, Varbobitis et al. [63] suggested a median total time of 7 min per examination. However, in the subgroup of patients with obesity, the total duration was prolonged (median 10 min) and exceeded 15 min in 5.5% of patients. In this study, 2D-SWE showed excellent reliability, as almost 98% of the examinations fulfilled the reliability criteria. An examination was considered to be reliable when at least five valid measurements were obtained.

## 5. Comparative Analysis of 2D-Shear Wave Elastography

### 5.1. Performances of 2D-SWE and Conventional US in Assessing Liver Fibrosis

2D-SWE has the advantage of undertaking both ultrasound examination and LSM at the same time, as it is integrated in a conventional ultrasound system. The performance of 2D-SWE and conventional US for the assessment of liver fibrosis and cirrhosis was evaluated by Zheng et al. [38]. In this study, results could not be obtained in only 1% of the patients, which was due to inability to optimally perform a breath hold and to liver atrophy. Moreover, 2D-SWE was superior to US in the diagnosis of both significant fibrosis (≥F2) and early cirrhosis (F4). The AUROC values were 0.86 for 2D-SWE and 0.73 for US for ≥F2 fibrosis, and 0.93 for 2D-SWE and 0.79 for US for F4 fibrosis. The sensitivity of 2D-SWE and US was 85.7% and 55.1% (≥F2), and 91.2% and 76.5% (F4), respectively. Similarly, the specificity of 2D-SWE and US was 79.3% and 85.5% (≥F2), and 79.7% and 71.4% (F4), respectively. The authors recommended that 2D-SWE be used after routine US in the assessment of liver fibrosis.

### 5.2. Comparison of the Usefulness of 2D-SWE and TE in People with Obesity

Among the non-invasive methods of assessing the severity of liver fibrosis, including TE, 2D-SWE has several advantages for people with obesity. The major limitation of TE is that results cannot be obtained in the approximately 20–25% of patients with a BMI greater than 30 kg/m^2^ [44,45]. Moreover, TE cannot be performed in patients with ascites, as elastic waves do not propagate through liquids [23,47]. On the other hand, the applicability of 2D-SWE is not limited by ascites or obesity [57]. Unlike TE where a vibration produces shear waves, 2D-SWE is integrated in a conventional ultrasound system that allows for visual control of the measurement location, avoiding vascular structures, studying both the left and right lobes of the liver, correlating elasticity with the observed tissue, and studying ROI and visualising the spatial distribution of fibrosis [39,49].

Nonetheless, it is worth noting that TE also allows detection and quantification of liver steatosis through controlled attenuation parameter (CAP) [64,65]. Similar to CAP from TE, a real-time B-mode ultrasound-based attenuation imaging (ATI) that quantifies liver steatosis has recently been introduced to 2D-SWE with promising results [66]. This allows assessment of both liver fibrosis and steatosis using 2D-SWE and ATI in a single ultrasound examination [66,67]. Practice guidelines [68,69] acknowledge TE as a clinically useful non-invasive tool for the identification of liver fibrosis in patients with NAFLD. As 2D-SWE is a relatively newly developed tool and follow-up data in patients with NAFLD are lacking, 2D-SWE has not yet been recommended in the current NAFLD guidelines [8]. The present findings highlight the need of further research to ascertain the usefulness of 2D-SWE.

### 5.3. Comparison of Diagnostic Performances of 2D-Shear Wave Elastography and Transient Elastography

Several studies have assessed the diagnostic performance of both 2D-SWE and TE for different fibrosis stages in patients with NAFLD (Table 2). Deffieux et al. [27] compared the accuracy of 2D-SWE with TE for staging and grading of fibrosis as assessed by liver biopsy. In this study, 2D-SWE had a success rate of 98%, and the diagnostic accuracy for fibrosis staging was similar between 2D-SWE and TE. The AUROC values of 2D-SWE and TE were 0.81 and 0.86 (≥F2), 0.80 and 0.82 (≥F3), 0.85 and 0.85 (F4), respectively. The optimal cut-off values for 2D-SWE were 8.9 and 10.2 kPa for ≥F2 and F4, respectively, and 6.9 and 12.8 kPa for TE.

A similar study by Cassinotto et al. [70] compared the diagnostic accuracy of 2D-SWE and TE in 291 patients with NAFLD (mean BMI 32.1 kg/m^2^). In comparison with patients with a BMI <30 kg/m^2^ (*n* = 116), proportions of reliable results were lower in BMI ≥30 kg/m^2^ (*n* = 175): 72.6% versus 90.5% for 2D-SWE and 70.9% versus 85.3% for TE. The AUROC values for 2D-SWE and TE were 0.86 and 0.82 (≥F2), 0.89 and 0.86 (≥F3), and 0.88 and 0.87 (F4), respectively. The cut-off values for 2D-SWE and TE for ruling out diseases with sensitivity ≥90% were similar: 6.3 and 6.2 kPa for the diagnosis of significant fibrosis (≥F2) and 10.5 and 9.5 kPa for the diagnosis of cirrhosis (F4), respectively. However, cut-off values for ruling in diseases with specificity ≥90% were lower for 2D-SWE than TE: 8.7 and 9.8 kPa for diagnosis of ≥F2 and 14.4 and 16.1 kPa for diagnosis of F4, respectively. Factors significantly associated with LSM results when using both 2D-SWE and TE were fibrosis stage, alkaline phosphatase, albumin, and waist circumference. The authors concluded that although obesity was associated with an increase in LSM failure, 2D-SWE provided a high value for the diagnosis of liver fibrosis in patients with NAFLD.

Furlan et al. [37] compared the diagnostic accuracy of 2D-SWE and TE in people with obesity (mean BMI 34.8 kg/m^2^) and NAFLD. For the diagnosis of significant fibrosis (≥F2), no statistically significant difference was observed between the AUROC values of 2D-SWE and TE, which were 0.80 and 0.77, respectively. A cut-off value of 7.2 kPa for 2D-SWE yielded sensitivity and specificity of 50% and 94%, respectively, and a cut-off value of 8.8 kPa for TE yielded sensitivity and specificity of 51% and 94%, respectively. In comparison, greater accuracy was found for both 2D-SWE and TE in diagnosing advanced fibrosis (≥F3), where the AUROC values were 0.89 for 2D-SWE and 0.86 for TE.

A recent study by Cassinotto et al. [71] evaluated the diagnostic performances of both 2D-SWE and TE in 577 patients with NAFLD (65.3% patients with BMI ≥30 kg/m^2^). For the diagnosis of advanced fibrosis (≥F3), highest accuracy was obtained with 2D-SWE (AUROC = 0.88) as compared to TE (AUROC = 0.82). The study also evaluated the diagnostic performances of a two-step strategy where 2D-SWE was followed by TE. Good performance for fibrosis detection was observed, with 82.3% accuracy, 88.3% sensitivity, and 80.9% specificity. Following 2D-SWE, 66.4% patients were found to need referral to a hepatologist, either due to a high risk of advanced fibrosis or the need to perform a second diagnostic step by TE. The study demonstrated that including 2D-SWE in multi-step strategies maintained excellent accuracy and decreased the need for liver biopsy.

The limitations of TE in patients with obesity have often been discussed. The use of TE with the M probe for assessment of liver fibrosis in patients with NAFLD (mean BMI 28.1 kg/m^2^) was evaluated by Imajo et al. [72]. The AUROC values for diagnosing ≥F2 and F4 were 0.82 and 0.92, respectively. In this study, TE was unsuccessful in assessing LSM in 10% of the patients, which could be attributed to various factors, including the presence of ascites and higher BMI. Attia et al. [73] evaluated the diagnostic accuracy of TE using an XL probe in 26 patients with obesity (mean BMI 36 kg/m^2^) suspected to have NAFLD. The AUROC values were 0.79 for ≥F2 and 0.92 for F4. Although results could not be obtained in 10% of the patients, the authors suggest that the use of an XL probe reduced the influence of BMI, steatosis, and steatohepatitis in LSM.

Similarly, Myers et al. [48] evaluated the diagnostic performance of the XL probe compared to the M probe in 276 patients with a BMI ≥28 kg/m^2^. In this study, LSM failure occurred in only 1.1% of patients with the XL probe, in comparison with 16% with the M probe. In patients with a BMI ≥40 kg/m^2^, failure rates of the XL and M probes were 5% and 59%, respectively. Similar results were reported by Poynard et al. [65], where the failure rate of TE using the M probe (8.2%) was considerably reduced to 2.8% by using the XL probe. In comparison, 2D-SWE had a very low failure rate of 0.9% in this study. Signal absence was considered a failure for TE, while no measurement and/or too low a signal was considered a failure for 2D-SWE, as a result of which, LSM could not be obtained. In patients with NAFLD and obesity, the findings indicate higher success rate for 2D-SWE, followed by TE using an XL probe and then an M probe.

## 6. Current Evidence and Future Perspectives

This review provides insight into the use of 2D-SWE in people with obesity for the assessment of liver fibrosis in NAFLD. Although TE is one of the most validated tools available, higher failure rates have been observed in people with obesity. 2D-SWE is emerging as a novel non-invasive diagnostic tool in people with obesity and severe obesity. As compared to TE, which is a separate device, 2D-SWE is integrated in a conventional ultrasound system, with the advantage of undertaking both ultrasound examination and LSM simultaneously. Hence, 2D-SWE may be particularly useful in various settings as it can be conducted as a part of routine ultrasound.

Several studies have reported a good diagnostic performance of 2D-SWE for the diagnosis of various stages of fibrosis in patients with NAFLD. 2D-SWE had a good accuracy and high success rate in patients with obesity and severe obesity who were at a high risk of developing NAFLD. However, exclusive focus on people with obesity was limited, which indicates the need for more research in this population. For the diagnosis of significant fibrosis (≥F2) and advanced fibrosis (≥F3), good to excellent diagnostic performances were reported. A few studies have compared the diagnostic accuracy of 2D-SWE with that of TE. The results indicate that 2D-SWE had a similar or better diagnostic performance and higher success rate than TE using both an M probe and an XL probe for assessing liver fibrosis in patients with obesity and NAFLD.

Use of 2D-SWE after routine US may be particularly useful for the diagnosis of significant fibrosis (≥F2) and early cirrhosis (F4). In patients with NAFLD, a two-step strategy using 2D-SWE followed by TE has shown good accuracy in advanced fibrosis (≥F3) detection. Multi-step strategies using 2D-SWE may also significantly reduce the need for liver biopsy. Due to the limited evidence of 2D-SWE in patients with NAFLD, it has not yet been recommended in the current NAFLD guidelines, which suggests the need of further research to ascertain its usefulness. Moreover, future research could assess the diagnostic performance of 2D-SWE along with markers of biochemical changes and give more clarity on the utility of non-invasive measures in assessing the progression of NAFLD in people with severe obesity.

## 7. Conclusions

With the rising incidence of obesity and severe obesity, the prevalence of NAFLD is also increasing at an alarming rate, and assessment is difficult due to the thicker subcutaneous adipose tissue layer. There is a need for the early detection and accurate diagnosis of NAFLD so that treatment can be instituted to prevent progression to advanced liver disease. There is a clinical need for a reliable and simple non-invasive method in the assessment of liver fibrosis as an alternative to liver biopsy. This review summarises the evidence from the current literature and suggests that 2D-SWE may be a promising alternative to liver biopsy as well as TE in the assessment of liver fibrosis in NAFLD, especially in people with obesity and severe obesity.

## Figures and Tables

**Table 1:** Diagnostic performance of 2D-SWE for different fibrosis stages in patients with non-alcoholic fatty liver disease (NAFLD).

Author	Year	Country	*n*	Mean BMI (kg/m^2^)	Fibrosis Stage	AUROC	Cut-Off (kPa)	Pooled Sensitivity	Pooled Specificity
Takeuchi et al. [61]	2018	Japan	71	29.2	≥F1	0.82	6.6	79%	67%
					≥F2	0.75	11.6	52%	44%
					≥F3	0.82	13.1	63%	57%
					F4	0.90	15.7	100%	82%
Herrmann et al. [62]	2018	Metanalysis 51 centres globally	156	31.2	≥F2	0.86	7.1	94%	52%
					≥F3	0.93	9.2	93%	81%
					F4	0.92	13.0	75%	88%
Jamialahmadi et al. [4]	2019	Iran	90	45.5	≥F1	0.77	5.6	71%	74%
					≥F2	0.72	6.6	72%	70%
					≥F3	0.77	6.8	80%	71%
					F4	0.70	6.8	100%	70%

METAVIR fibrosis stage: F1: mild fibrosis; F2: significant fibrosis; F3: severe fibrosis; F4: cirrhosis. n: sample size. BMI: body mass index. AUROC: area under receiver operating characteristics curve.

**Table 2:** Diagnostic performance of 2D-SWE and transient elastography (TE) for different fibrosis stages in patients with NAFLD.

Author	Year	Country	*n*	Mean BMI (kg/m^2^)	Technique	Fibrosis Stage	AUROC	Cut-Off (kPa)	Pooled Sensitivity	Pooled Specificity
Deffieux et al. [27]	2015	France	120	24.2	2D-SWE	≥F2	0.81	8.9	77%	79%
						≥F3	0.80	9.1	85%	72%
						F4	0.85	10.2	83%	76%
					TE	≥F2	0.86	6.9	74%	87%
						≥F3	0.82	7.4	78%	81%
						F4	0.85	12.8	73%	88%
Cassinotto et al. [70]	2016	France	291	32.1	2D-SWE	≥F2	0.86	6.3	90%	50%
								8.7	71%	90%
						≥F3	0.89	8.3	91%	71%
								10.7	71%	90%
						F4	0.88	10.5	90%	72%
								14.4	58%	90%
					TE	≥F2	0.82	6.2	90%	45%
								9.8	60%	90%
						≥F3	0.86	8.2	90%	61%
								12.5	57%	90%
						F4	0.87	9.5	92%	62%
								16.1	65%	90%
Furlan et al. [37]	2019	United States	62	34.8	2D-SWE	≥F2	0.80	7.2	50%	94%
						≥F3	0.89	8.0	71%	92%
					TE	≥F2	0.77	8.8	51%	94%
						≥F3	0.86	10.5	50%	92%
Cassinotto et al. [71]	2020	France	577	31.8	2D-SWE	≥F3	0.88	8.0	>90%	-
								10.5	-	>90%
					TE	≥F3	0.82	6.8	>90%	-
								12.0	-	>90%

METAVIR fibrosis stage: F1: mild fibrosis; F2: significant fibrosis; F3: severe fibrosis; F4: cirrhosis. n: sample size. BMI: body mass index. AUROC: area under receiver operating characteristics curve.

## Data Availability

Data is contained within the article.
